# Pregnancy and neonatal outcomes following time-lapse versus conventional embryo culture: a retrospective cohort study

**DOI:** 10.3389/fendo.2026.1722638

**Published:** 2026-02-11

**Authors:** Wei Li, Miaomiao Xin, Xia Xue, Tingting He, Juanzi Shi

**Affiliations:** Assisted Reproduction Center, Northwest Women’s and Children’s Hospital, Xi’an, China

**Keywords:** conventional embryo culture, neonatal outcomes, pregnancy outcomes, PSM, time-lapse culture

## Abstract

**Objective:**

To compare pregnancy and neonatal outcomes between time-lapse and conventional embryo culture in frozen embryo transfer (FET) cycles.

**Design:**

Retrospective cohort study.

**Setting:**

Tertiary-care academic medical center.

**Patients:**

This retrospective cohort study included 15,697 women who underwent single blastocyst transfer during their first FET cycle from January 2017 to December 2023.

**Intervention(s):**

None.

**Main Outcome Measure(s):**

The pregnancy outcomes and the neonatal outcomes.

**Result(s):**

Our study demonstrated that pregnancy outcomes were comparable between the time-lapse embryo culture group and the conventional embryo culture group, including biochemical pregnancy (relative risk [RR] = 1.02; 95% confidence interval [CI], 0.92–1.13), clinical pregnancy (RR = 1.07; 95% CI: 0.96–1.18), ectopic pregnancy (RR = 0.65; 95% CI: 0.37–1.17), miscarriage (RR = 0.99; 95% CI: 0.86–1.16), live birth (RR = 1.07; 95% CI: 0.98–1.18) and multiple live births (RR = 0.94; 95% CI: 0.57–1.54). Additionally, no statistically significant differences were observed in neonatal outcomes, including cesarean delivery, male gender, very preterm birth, preterm birth, very low birth weight, low birth weight) or high birth weight. Similarly, there were no statistically significant differences in either gestational age or birth weight between the two groups.

**Conclusion(s):**

Our study demonstrated that time-lapse embryo culture yields comparable pregnancy and neonatal outcomes to conventional embryo culture, suggesting that this technology is both effective and safe for embryo incubation.

## 1 Introduction

Embryo culture plays a vital role in the procedure of *in vitro* fertilization (IVF). In traditional IVF procedures, developing embryos are removed from the incubator for morphological assessment at specific time points (1). However, removing embryos from the incubator for morphological assessment may negatively affect embryo development due to fluctuations in temperature, gas concentrations, and pH levels of the culture medium (2). Furthermore, embryo handling increases the risk of cellular damage and embryo loss (3). In recent years, an increasing number of IVF laboratories utilize time-lapse monitoring for embryo assessment. In time-lapse incubators, embryos are continuously monitored by built-in cameras that capture images at predefined intervals, thereby eliminating the need to remove embryos from the incubator (4, 5). This minimizes the potential negative effects of environmental fluctuations and human handling on embryo development. Therefore, time-lapse incubation is widely regarded as a safe and effective technology for monitoring human embryos.

However, it remains unclear whether time-lapse incubation improves pregnancy and neonatal outcomes compared to conventional incubation. A randomized controlled trial (RCT) conducted by Insua MF et al., including 856 women who underwent embryo or blastocyst transfer, reported no differences in the live birth rate between the two groups (6). Additionally, a recent large-scale RCT involving women who underwent fresh single embryo transfer demonstrated that the ongoing pregnancy rates were comparable between the two groups (4). In contrast, a RCT using Day 3 double embryo transfer showed that time-lapse incubation significantly improved clinical pregnancy rates compared to conventional incubation (65.2% vs. 77.8%), though live birth rates remained comparable (63.9% vs. 55.3%). Notably, the conventional incubation group exhibited a higher incidence of secondary infertility compared to the time-lapse group (40.2% vs. 37.6%) (7). In addition, there is ongoing debate regarding the neonatal outcomes associated with time-lapse incubation. Most studies have shown no significant differences between time-lapse incubation and conventional incubation, while two other studies have suggested that time-lapse incubation is associated with improved neonatal outcomes (6–10). This controversy arises from the lack of sufficient high-quality evidence to support the routine use of time-lapse incubation.

Therefore, to compare pregnancy and neonatal outcomes between time-lapse and conventional embryo culture, we included only women who underwent single blastocyst transfer during their first frozen embryo transfer (FET) cycle. Given the significant influence of baseline characteristics on clinical outcomes, propensity score matching (PSM) was performed to balance the baseline characteristics between the two groups.

## 2 Methods

### 2.1 Study design and participants

This retrospective cohort study was conducted at the Center for Assisted Reproductive Technology of Northwest Women’s and Children’s Hospital, People’s Republic of China, from January 2017 to December 2023. The study included women who underwent single blastocyst embryo transfer in their first FET cycle. The exclusion criteria were as follows: [1] oocyte donation cycles; [2] preimplantation genetic testing cycles; [3] women with uterine malformations; [4] women with endometrial polyps, submucosal fibroids, intrauterine adhesions; and [5] cycles with incomplete data due to loss to follow-up. Ultimately, a total of 15,697 women were included in the study, with 3,495 allocated to the time-lapse culture group and 12,202 to the conventional culture group. This study was approved by the Ethics Committee of the Northwest Women’s and Children’s Hospital (number 2023003). Informed consent was obtained from each patient.

### 2.2 IVF/Intracytoplasmic sperm injection protocols and embryo culture

The ovarian stimulation protocols utilized in this study have been described in detail in a previous study, with a mean female age of 30.40 ± 4.06 years (11). Human chorionic gonadotropin (HCG) was administered when at least two follicles reached a mean diameter of 17 mm. Oocyte retrieval was performed 36–38 hours later under transvaginal ultrasound guidance. Following identification under a stereomicroscope, cumulus–oocyte complexes (COCs) were initially washed in G-MOPS Plus medium (Vitrolife, Sweden), then transferred to G-IVF medium (Vitrolife, Sweden) for incubation for 2–3 hours. For patients undergoing IVF, COCs were placed in a 4-well culture dish containing G-IVF Plus medium, with a final sperm concentration of approximately 50,000 motile spermatozoa per COC. After 4–6 hours of co-incubation of sperm and oocytes, cumulus cells surrounding the oocytes were mechanically removed using a denudation pipette, and the extrusion of the second polar body was observed and documented. For patients undergoing ICSI, cumulus cells were removed using hyaluronidase, and ICSI was performed within 1–2 hours later. Zygotes were subsequently transferred to G1 Plus medium (Vitrolife, Sweden) and cultured individually in either conventional or time-lapse incubators. The gas mixing in both incubator systems was identical, with 5% oxygen (O_2_) and 6% carbon dioxide (CO_2_) maintained at 37 °C in a humidified environment. On Day 3 of embryo culture, the medium was changed from G1 Plus to G2 Plus medium (Vitrolife, Sweden).

### 2.3 Blastocyst morphological evaluation, vitrification and warming

Blastocyst morphology was assessed using the Gardner scoring system, which evaluates the degree of blastocoel expansion, inner cell mass (ICM) development, and trophectoderm (TE) appearance (12). Good-quality embryos were classified as blastocysts graded as AA, AB, BA and BB with an expansion grade of ≥ 3, while poor-quality embryos were categorized as AC, CA, BC, CB and CC with an expansion grade of ≥ 3. The Kitazato vitrification kit (Kitazato, Japan) was used for both vitrification and warming. Vitrification solution (VS) (Kitazato, Japan) containing blastocysts was placed onto the surface of a Cryotop strip (Kitazato, Japan) and immediately submerged in liquid nitrogen. For warming, the Cryotop strip was quickly removed from liquid nitrogen and transferred to a pre-equilibrated thawing solution (TS) (Kitazato, Japan) for 1 minute at 37 °C. The strip was then sequentially washed in dilution solution (DS) (Kitazato, Japan), washing solution 1 (WS1) (Kitazato, Japan), and washing solution 2 (WS2) at room temperature. The transfer of blastocysts was performed 2 hours after warming.

### 2.4 Outcome measures

Biochemical pregnancy was defined as a serum β-hCG level ≥ 20 IU/L on Day 12 after blastocyst transfer. Clinical pregnancy was defined as the presence of at least one intrauterine gestational sac visualized by transvaginal ultrasound. Ectopic pregnancy was defined as the observation of a gestational sac outside the uterine cavity. Miscarriage was defined as fetal loss occurring < 24 weeks of gestation, whereas live birth was defined as the delivery of at least one live-born infant ≥ 24 weeks of gestation (13). In addition, neonatal outcomes were also investigated in this study, including very preterm birth (VPTB, < 32 weeks of gestation), preterm birth (PTB, < 37 weeks of gestation), very low birth weight (VLBW, < 1500 g), low birth weight (LBW, < 2500 g), and high birth weight (HBW, > 4000 g) (14).

### 2.5 Statistical analysis

All analyses were performed using SPSS software version 22.0 (SPSS Inc., Chicago, USA). Continuous variables are presented as mean ± standard deviation (SD), while categorical data are presented as frequencies and percentages. Considering the potential impact of baseline characteristics on pregnancy and neonatal outcomes, PSM without replacement was performed to balance the two groups, using a 1:1 match ratio and a caliper width of 0.2 standard deviations (15). The following baseline characteristics were included in the construction of the propensity score: maternal age, maternal body mass index (BMI), endometrial thickness (EMT), infertility duration, infertility type, infertility cause, endometrial preparation protocols, fertilization type, and embryo quality. The balance of baseline characteristics between the two groups was evaluated using standardized differences, with values < 10% indicating adequate covariate balance (16).

After PSM, generalized linear models (GLMs) were used to compare pregnancy and neonatal outcomes between the two groups, with the matched group specified as the random effect to account for within-pair correlation. Specifically, for categorical variables, a log-binomial regression model was applied when the incidence was ≥ 5%, whereas a log-Poisson regression model was used when the incidence was < 5%. For continuous outcome variables, a normal-identity regression model with an identity link function was employed to estimate mean differences. The results are presented as risk ratios (RRs) or mean differences, along with their corresponding 95% confidence intervals (CIs).

## 3 Results

### 3.1 Participant characteristics

**Figure 1:**
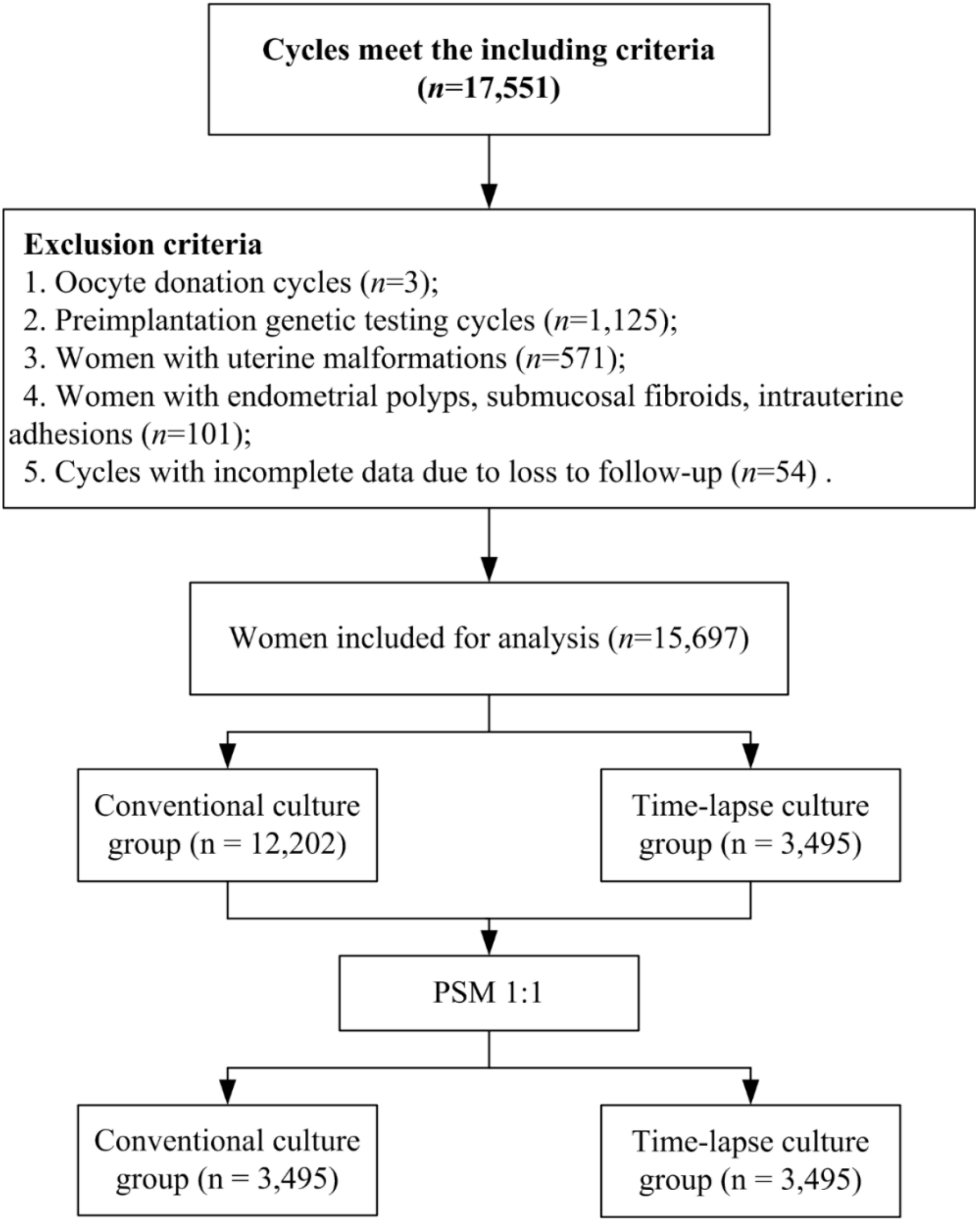
Flow chart of the study. *PSM* propensity matching .

This retrospective cohort study included 15,697 women who underwent single blastocyst transfer in their first FET cycle. The participants were divided into two groups: the time-lapse culture group (n = 3,495) and the conventional culture group (n = 12,202) (**Figure 1**). The baseline characteristics of the women before and after PSM are presented in **Table 1**. Before PSM, all standardized differences were > 10%, except for maternal BMI, EMT, infertility duration, infertility type, and infertility cause. To balance the baseline characteristics, PSM was performed to match 3,495 women in the time-lapse culture group with 3,495 women in the conventional culture group. After PSM, all standardized differences were found to be less than 10%, indicating well-matched patients with similar baseline characteristics between the two groups.

**Table 1:** Baseline characteristics according to incubator culture type before and after PSM.

Characteristics	Before PSM			After PSM		
	Conventional culture group (*n* = 12,202)	Time-lapse culture group (*n* = 3,495)	Standardized difference (%)	Conventional culture group (*n* = 3,495)	Time-lapse culture group (*n* = 3,495)	Standardized difference (%)
Maternal age (y)	31.40 ± 4.00	30.93 ± 3.66	12	30.98 ± 3.58	30.93 ± 3.66	2
Maternal BMI (kg/m2)	22.68 ± 3.41	22.71 ± 3.42	1	22.62 ± 3.37	22.71 ± 3.42	3
EMT (mm)	10.53 ± 1.73	10.53 ± 1.71	0	10.54 ± 1.70	10.53 ± 1.71	0
Infertility duration (y)	3.39 ± 2.38	3.41 ± 2.35	1	3.43 ± 2.37	3.41 ± 2.35	1
Infertility type, n (%)			4			1
Primary	6,528 (53.50)	1,948 (55.74)		1,959 (56.05)	1,948 (55.74)	
Second	5,674 (46.50)	1,547 (44.26)		1,536 (43.95)	1,547 (44.26)	
Infertility cause, n (%)			6			5
Male	1,899 (15.56)	601 (17.20)		642 (18.37)	601 (17.20)	
Female	7,997 (65.54)	2,192 (62.72)		2,201 (62.98)	2,192 (62.72)	
Both	1,437 (11.78)	441 (12.62)		431 (12.33)	441 (12.62)	
Unknown	869 (7.12)	261 (7.47)		221 (6.32)	261 (7.47)	
Endometrial preparation protocols, n (%)			11			6
Natural cycle	3,156 (25.86)	822 (23.52)		871 (24.92)	822 (23.52)	
HRT	6,936 (56.84)	2,164 (61.92)		2,070 (59.23)	2,164 (61.92)	
GnRH agonist-HRT	2,110 (17.29)	509 (14.56)		554 (15.85)	509 (14.56)	
Fertilization type, n (%)			15			2
IVF	10,074 (82.56)	2,671 (76.42)		2,698 (77.20)	2,671 (76.42)	
ICSI	2,128 (17.44)	824 (23.58)		797 (22.80)	824 (23.58)	
Number of good embryo transferred, n (%)			25			1
0	4,481 (36.72)	882 (25.24)		861 (24.64)	882 (25.24)	
≥1	7,721 (63.28)	2,613 (74.76)		2,634 (75.36)	2,613 (74.76)	

*Note*: Values are presented as mean ± standard deviation or number (percentage).

PSM, propensity matching; BMI, body mass index; EMT, endometrial thickness; HRT, hormone replacement therapy; GnRH, gonadotropin-releasing hormone; IVF, in vitro fertilization; ICSI, intracytoplasmic sperm injection.

### 3.2 Pregnancy and neonatal outcomes

**Table 2** showed the pregnancy outcomes. Compared with the conventional culture group, the time-lapse culture group showed no significant differences in biochemical pregnancy (RR = 1.02; 95% CI: 0.92–1.13), clinical pregnancy (RR = 1.07; 95% CI: 0.96–1.18), ectopic pregnancy (RR = 0.65; 95% CI: 0.37–1.17), and miscarriage (RR = 0.99; 95% CI: 0.86–1.16). Similarly, live birth (RR = 1.07; 95% CI: 0.98–1.18) and multiple live births (RR = 0.94; 95% CI: 0.57–1.54) were also comparable between the two groups.

**Table 2:** Pregnancy outcomes according to incubator culture type in the propensity-score-matched cohort.

Characteristics	Conventional culture group (*n* = 3,495)	Time-lapse culture group (*n* = 3,495)	RR (95% CI)
Biochemical pregnancy, n (%)	2,537 (72.59)	2,550 (72.96)	1.02 (0.92, 1,13)
Clinical pregnancy, n (%)	2,298 (65.75)	2,347 (67.15)	1.07 (0.96, 1.18)
Ectopic pregnancy, n (%)	29 (0.83)	19 (0.54)	0.65 (0.37, 1.17)
Miscarriage, n (%)	380 (10.87)	379 (10.84)	0.99 (0.86, 1.16)
Live birth, n (%)	1,889 (54.05)	1,949 (55.77)	1.07 (0.98, 1.18)
Multiple live births, n (%)	32 (1.69)	31 (1.59)	0.94 (0.57, 1.54)

Values are presented as number (percentage).

RR, relative risk; CI, confidence interval.

**Table 3** showed the neonatal outcomes. No significant differences were observed between the time-lapse and conventional culture groups in cesarean delivery (RR = 0.94; 95% CI: 0.81–1.08), male gender (RR = 1.07; 95% CI: 0.94–1.21), VPTB (RR = 0.77; 95% CI: 0.43–1.40), PTB (RR = 0.85; 95% CI: 0.68–1.06), VLBW (RR = 0.97; 95% CI: 0.46–2.04), LBW (RR = 0.73; 95% CI: 0.56–0.95) or HBW (RR = 0.90; 95% CI: 0.70–1.15). Similarly, gestational age (mean difference = 0.07, 95% CI: –0.04 to 0.18) and birth weight (mean difference = 3.14, 95% CI: –30.25 to 36.54) did not differ significantly between the two groups. Similarly, there were no statistically significant differences in either gestational age (mean difference = 0.07 weeks; 95% CI, –0.04 to 0.18) or birth weight (mean difference = 3.14 g; 95% CI, –30.25 to 36.54) between the two groups.

**Table 3:** Neonatal outcomes according to incubator culture type in the propensity-score-matched cohort.

Characteristics	Conventional culture group (*n* = 3,495)	Time-lapse culture group (*n* = 3,495)	RR/Mean difference (95% CI)
Cesarean delivery, n (%)	1,417 (75.01)	1,438 (73.78)	0.94 (0.81, 1.08)
Male gender, n (%)	1,039 (55.00)	1,104 (56.64)	1.07 (0.94, 1.21)
Very preterm birth, n (%)	25 (1.32)	20 (1.03)	0.77 (0.43, 1.40)
Preterm birth, n (%)	182 (9.63)	161 (8.26)	0.85 (0.68, 1.06)
Very low birth weight (<1,500 g), n (%)	14 (0.74)	14 (0.72)	0.97 (0.46, 2.04)
Low birth weight (<2,500 g), n (%)	131 (6.93)	100 (5.13)	0.73 (0.56, 0.95)
High birth weight (>4,000 g), n (%)	138 (7.31)	129 (6.62)	0.90 (0.70, 1.15)
Gestational age (week)	38.80 ± 1.84	38.87 ± 1.69	0.07 (-0.04, 0.18)
Birth weight (g)	3,320.88 ± 546.23	3,324.02 ± 509.07	3.14 (-30.25, 36.54)

Values are presented as mean ± standard deviation or number (percentage).

RR, relative risk; CI, confidence interval.

## 4 Discussion

The present retrospective cohort study compared pregnancy and neonatal outcomes between time-lapse and conventional embryo culture in women undergoing single blastocyst transfer during their first FET cycle. The results showed that pregnancy outcomes were comparable between the two groups, including biochemical pregnancy, clinical pregnancy, ectopic pregnancy, miscarriage, live birth, and multiple live births. Additionally, no statistically significant differences were observed in neonatal outcomes, including cesarean delivery, male gender, VPTB, PTB, VLBW, LBW, HBW, gestational age, and birth weight.

Our results demonstrated that pregnancy outcomes were comparable between the two groups, consistent with findings from previous studies. A RCT conducted by Zhang et al., based on 1,224 women who underwent transfer of one or two cleavage-stage embryos on day 3, reported no statistically significant difference in live birth rates between the groups (17). Additionally, a recent large-scale RCT involving women undergoing fresh single embryo transfer demonstrated that the pregnancy outcomes were comparable between the two groups (4). A retrospective study also reported that miscarriage, ectopic pregnancy, and live birth were comparable between the two groups (8). However, a cohort study by Meng Q et al., involving 139 women who underwent single embryo transfer, reported that both clinical pregnancy and live birth rates were significantly lower in the time-lapse culture group compared with the conventional embryo culture group (1). However, when the analysis was restricted to FET cycles, these differences were no longer observed (1). These inconsistent findings may be attributable to population heterogeneity, such as differences in cycle types (fresh vs. FET), embryo stage (cleavage-stage vs. blastocyst), and the number of embryos transferred. To improve the reliability and consistency of the present study, only women who underwent single blastocyst transfer during their first FET cycle were included.

We also observed no statistically significant differences in neonatal outcomes between the two groups, including cesarean delivery, male gender, VPTB, PTB, VLBW, LBW, HBW, gestational age, and birth weight. Similarly, Ma BX et al. reported no significant differences in PTB, gestational age, birth weight, or sex ratio between the group (8). A recent large-scale study by Ahlström A et al., involving 7,379 deliveries following fresh embryo transfer, demonstrated that there were no significant differences between time-lapse and conventional embryo culture in terms of PTB (odds ratio [OR], 1.11; 95% CI, 0.87–1.41) or LBW (adjusted odds ratio [aOR], 0.86; 95% CI, 0.66–1.14) (10). In contrast, Mascarenhas M et al. reported that, compared to the conventional embryo culture group, neonatal outcomes in the time-lapse group were associated with a lower risk of VPTB and LBW in FET cycles (9). However, it is worth noting that this study included only a limited number of participants, resulting in a total of 282 live births.

Time-lapse embryo culture offers two main advantages. First, it provides continuous and detailed morphokinetic information on embryo development, enabling more refined assessment compared with conventional culture (5). Second, it minimizes fluctuations in humidity, temperature, pH, and gas concentrations by eliminating the need to remove embryos from the incubator (5). Previous studies have shown that the time-lapse system alleviates oxidative stress and promotes early embryonic development (18, 19). However, it may not be clinically significant enough to substantially impact the outcomes. This could be due to both the inherent resilience of embryos to transient environmental perturbations and the effectiveness of standard laboratory protocols in minimizing disruptions during brief exposures outside the incubator (20). In addition, light exposure during time-lapse imaging may have potential adverse effects on embryos, as embryos are subjected to illumination at intervals as short as every 10 minutes. Although the total ultraviolet radiation is relatively low, the possibility of phototoxic effects cannot be entirely excluded (21). Therefore, embryo culture using time-lapse yields comparable pregnancy and neonatal outcomes compared to conventional embryo culture.

Current evidence suggests no significant differences in perinatal or maternal outcomes between time-lapse and conventional culture methods, aligning with our findings (6, 8). While time-lapse systems offer advanced imaging and continuous monitoring, they require a higher initial investment and entail greater technical complexity, which may limit their adoption in resource-constrained settings or smaller laboratories (22). In contrast, conventional incubators remain more cost-effective and widely accessible due to their lower operational and maintenance demands. Ultimately, the choice between these systems should be guided by the specific clinical or research priorities, as well as the available infrastructure.

Notably, this study has several strengths. The primary strength lies in the rigorous participant selection, as only women who underwent single blastocyst transfer during their first FET cycle were included. Additionally, to ensure comparability between groups, PSM was performed, thereby enhancing the robustness and credibility of the findings. Furthermore, the large sample size provided sufficient statistical power to evaluate both pregnancy and neonatal outcomes, offering important evidence for future research and clinical decision-making.

However, this study also has several limitations. First, the findings may not be generalizable to cleavage-stage embryo transfer strategies or to older patients with a poor prognosis. Second, this study did not differentiate between elective and medically indicated cesarean deliveries. Thirdly, the study data were derived from a single medical center, which may introduce selection bias and limit external validity. Therefore, future multi-center randomized controlled trials (RCTs) are warranted to validate and extend the applicability of our findings.

## 5 Conclusion

In conclusion, our study demonstrated that embryo culture using time-lapse yields comparable pregnancy and neonatal outcomes compared to conventional embryo culture, suggesting that this technology is effective and safe for embryo incubation. However, long-term follow-up studies are necessary to further confirm the safety of time-lapse incubation.

## Data Availability

The raw data supporting the conclusions of this article will be made available by the authors, without undue reservation.
